# Virtual Environment for Manipulating Microscopic Particles With Optical Tweezers

**DOI:** 10.6028/jres.108.026

**Published:** 2003-08-01

**Authors:** Yong-Gu Lee, Kevin W. Lyons, Thomas W. LeBrun

**Affiliations:** National Institute of Standards and Technology, Gaithersburg, MD 20899-0001

**Keywords:** nanoscale assembly, nanotechnology, optical tweezers, virtual reality

## Abstract

In this paper, virtual reality techniques are used to define an intuitive interface to a nanoscale manipulation device. This device utilizes optical methods to focus laser light to trap and reposition nano-to-microscopic particles. The underlying physics are simulated by the use of Lagrange mechanics. A unique control method for the manipulation of the particles is also provided. The user can naturally grab and steer the particles. Behind the scene, a complex computation is performed to find the new location of the potential field induced by the laser beam that would move the particles accordingly. Haptic feedback is used to constrain the steering motion within the physical capability of the potential field.

## 1. Introduction

The development of nanotechnology [1] has generated a great deal of scientific interest, but the promise of nanotechnology will be realized only by manufacturing commercial products from assemblies of atoms, molecules and nanoscale components. While nanoscale measurement and fabrication techniques are advancing rapidly, few effective techniques for manipulation of nanoscale objects are available. Manipulation of nanoscale objects can be achieved using a scanning probe microscope, such as an AFM (Atomic Force Microscope) or an STM (Scanning Tunneling Microscope). Guthold et al. [2] performed rolling and sliding of carbon nanotubes using an AFM. Eigler and Scheweizer [3] used an STM to position individual xenon atoms on a single-crystal nickel surface, but these tools do not yet offer very meticulous control of objects (e.g., the ability to grasp and release). Laser beams can also be used to trap and manipulate small particles. A laser apparatus, called OT (optical tweezers) [4], provides the user with a noncontact method for manipulating objects that can be applied to viruses, bacteria, living cells and synthetic micro and nanoscale particles.

Each method has strengths and weaknesses. The AFM can be used to manipulate a larger set of objects, and the relative forces required to manipulate the particles can be more easily derived. The disadvantage is that it is generally less accurate than the STM, and since the AFM can directly contact the sample, considerations of sharpness and wear of the tool tip must be accounted for. The STM, being non-contact, has little tool wear, yet manipulation is limited to conducting and semiconductor materials and typically will occur under vacuum conditions. The strength of OT is that they are well suited for manipulating biological objects and refractive and metallic particles, yet it is usually used in solution. The AFM and STM are generally limited to two dimensions with a very limited third dimension (sometimes referred to 2.5 dimensions) whilst OT can work in three dimensions. It is also interesting to note that AFM and STM can work both in imaging and manipulation mode, but OT has primarily been used for manipulation, although 3D imaging has been reported [5]. All these techniques require complex driving electronics under sophisticated computer control.

The operation of nanoscale manipulators can be done through tele-operation or automatic manipulation. In the former approach, a human operator acts as a part of the control loop and issues commands by judgments based on various forms of sensory cues. The sensory cues can be audio, visual and haptic. In the latter approach, the computer program makes autonomous decisions based on an underlying set of constraints or rules to perform the predetermined manipulation scenario. In developing these constraints and rules, one must have some understanding of nanoscale dynamics, but this is still an emerging field of research. This is why tele-operation is gaining favor for certain user interfaces. Tele-operation needs to virtually present the nanoscale environment to the operator such that the operator can be aware of the target objects and the surroundings. Various techniques in virtual reality can be used to enhance this human-in-the-loop control system by letting the user feel immersed in the environment. Among these techniques, haptic display plays a vital role for the following reason.

One of the most challenging problems in nanoscale manipulation is that real-time image sensing of nanoscale artifacts is difficult. The imaging sampling time of an AFM and STM can take seconds, or possibly minutes, depending on the scan rate and the sampling range, and cannot be done simultaneously with the manipulation. Thus the only real-time feedback occurs with the AFM by way of the force imparted on the probe when sensing nanoscale objects (contact or non-contact modes) or by the STM current fluctuations that can be correlated to height of the tip above the surface. The user feedback occurs through linking the AFM force or STM height measurement directly to a haptic device using an appropriate amplification routine. This need for real-time exploration is what attracts researchers to develop man-machine interfaces for nanoscale manipulation that use haptic display technologies. Some of the work is described below.

Hollis et al. [6] demonstrated that atomic-scale landscapes could be explored and felt with the hand in real time by interfacing a haptic feedback device with an STM. Their haptic device is based on Lorentz magnetic levitation. Sitti and Hashimoto [7] used an AFM with a one degree of freedom haptic device to sense nanoscale forces. They demonstrated two-dimensional positioning of micrometer sized latex particles. Guthold et al. [2] used a force feedback device coupled with an AFM tip to haptically sense and manipulate carbon nanotubes, DNA and viruses. All of these research efforts used a graphical display in addition to the haptic display. The topographical data used for the graphical display of the nanoscale objects were acquired by using the probe in the imaging mode that was also used in the manipulation mode. The limitation of this method is that while manipulating the specimen, the graphical display is static and requires additional scans to see the result of the manipulation.

With OT, typical manipulation can be done through a two-dimensional pointing device such as a mouse or joystick. Usually the manipulation is done in noncontact mode, so there is no direct force that can be rendered on a haptic display. Because of the limited ability of traditional optical microscopy to resolve nanometer-scale structures, OT, unlike AFM or STM, must use other means and synthesize virtual sensory inputs to the operator. This paper presents the work on developing an intuitive interface for manipulating particles through OT with the use of VR (Virtual Reality).

## 2. Approach

The Lagrange equation that serves as the foundational model for the trajectory of a particle exposed to a potential field is first introduced. The potential field is an abstraction of the potential energy field that is induced by the laser beam. Note the potential field depends on both the laser and the particle. For example, if the geometry of the particle changes, the potential field is no longer applicable. Then the equation of potential field position that can be used to steer a particle is derived. Lastly, an approach in constraining the steering motion within the physical capability of the optical laser instrument through the use of haptic feedback is presented.

### 2.1 Modeling of Optical Tweezers Physics

The time required to model the physics associated with the optical tweezers instrument depends on the objective of the work and the accuracies required. Maxwell's equations can be used at the fine level, but geometric optics calculations can give sufficient explanations for most cases [8]. The approach described in this paper goes one step further and assumes that the potential energy can be represented by an explicit spatial function. For the convenience of implementation only two-dimensional space and the motions within this space is discussed, but this involves no loss of generality for a sphere trapped in a cylindrically symmetric beam.

As an example, assume one has a spherical particle of radius *r*, mass *m* and with a homogeneous material distribution. The form of the trapping potential will depend on the size and properties of both the beam and the particle, as well as the index of refraction of the surrounding medium. To focus on the visualization of trapped particles, a simple potential well with a Gaussian distribution is assumed. Assume that due to the laser beam, the approximate potential energy of this particle can be given by

$$P=-P_{0}e^{-\ln(2)\left(\frac{x^{2}}{a^{2}}+\frac{y^{2}}{b^{2}}\right)},$$ (1)

where *P*_0_ is the well depth, *x* and *y* are the two axes perpendicular to the laser path, and *a* and *b* are the beam waist dimensions. The factor 2 in Eq. (1) normalizes the potential so that its value at *x* = *a*, *y* = 0 or at *x* = 0, *y* = *b* is equal to $-\frac{P_{0}}{2}$. The potential field given in Eq. (1) can be realized by dithering the laser beam or through the use of multiple laser beams. Equation (1) can be used to describe a spherical potential when *a* and *b* are the same or a line potential when one of the beam waist dimensions is much greater than the other. Assuming there is no change in the rotational speed of the particle, the kinetic energy of this particle can be given by

$$T=\frac{1}{2}m\left({\dot{x}}^{2}+{\dot{y}}^{2}\right).$$ (2)

From Eqs. (1) and (2), Lagrangian equations of motion [9] allows us to predict the behavior of the particle trajectory. The behavior is expressed as a system of n second-order differential equations,

$$\frac{d}{dt}\left(\frac{\partial T}{\partial {\dot{q}}_{i}}\right)-\frac{\partial T}{\partial q_{i}}+\frac{\partial P}{\partial q_{i}}=Q_{i},$$ (3)

where *q*_0_,*q*_1_,⋯*q_n_*_–1_ and ${\dot{q}}_{0},{\dot{q}}_{1},\cdots ,{\dot{q}}_{n-1}$ are the generalized coordinates and the time derivatives of these coordinates. *Q*_0_,*Q*_1_,⋯,*Q_n_*_–1_ are generalized forces.

In Cartesian coordinates, Eq. (3) expands to

$$\begin{array}{l} \frac{d}{dt}\left(\frac{\partial T}{\partial \dot{x}}\right)-\frac{\partial T}{\partial x}+\frac{\partial P}{\partial x}=F_{x},\\ \frac{d}{dt}\left(\frac{\partial T}{\partial \dot{y}}\right)-\frac{\partial T}{\partial y}+\frac{\partial P}{\partial y}=F_{y}, \end{array}$$ (4)

where *F_x_* and *F_y_* are external forces to the system. For example, viscous forces can be used as the external forces. However, for simplicity, they are not considered in the calculations.

Substituting the *T* in Eq. (2) and the *P* in Eq. (1) to Eq. (4) leads to

$$\begin{array}{l} \frac{d}{dt}\left\{\frac{\partial }{\partial \dot{x}}\left[\frac{1}{2}m\left({\dot{x}}^{2}+{\dot{y}}^{2}\right)\right]\right\}-\frac{\partial }{\partial x}\left[\frac{1}{2}m\left({\dot{x}}^{2}+{\dot{y}}^{2}\right)\right]\\ +\frac{\partial }{\partial x}\left(-P_{0}e{-}^{-\ln(2)\left(\frac{x^{2}}{a^{2}}+\frac{y^{2}}{b^{2}}\right)}\right)=F_{x},\\ \frac{d}{dt}\left\{\frac{\partial }{\partial \dot{y}}\left[\frac{1}{2}m\left({\dot{x}}^{2}+{\dot{y}}^{2}\right)\right]\right\}-\frac{\partial }{\partial y}\left[\frac{1}{2}m\left({\dot{x}}^{2}+{\dot{y}}^{2}\right)\right]\\ +\frac{\partial }{\partial y}\left(-P_{0}e{-}^{-\ln(2)\left(\frac{x^{2}}{a^{2}}+\frac{y^{2}}{b^{2}}\right)}\right)=F_{y}. \end{array}$$ (5)

Rearranging the above leads to

$$\begin{array}{l} m\ddot{x}-\frac{-2\ln\left(2\right)P_{0}x}{a^{2}}e^{-\ln(2)\left(\frac{x^{2}}{a^{2}}+\frac{y^{2}}{b^{2}}\right)}=F_{x},\\ m\ddot{y}-\frac{-2\ln\left(2\right)P_{0}y}{b^{2}}e^{-\ln(2)\left(\frac{x^{2}}{a^{2}}+\frac{y^{2}}{b^{2}}\right)}=F_{y}. \end{array}$$ (6)

Equation (6) can not be solved analytically, and a fourth-order Runge-Kutta method for solving the differential equation is used. With the help of Eq. (6), the trajectory of a particle can be simulated. What is more interesting is that one can solve for the new location of the laser trap (center of the potential field) such that it would influence the particle to move in a certain direction within a specific time lapse. This can be used to steer the particle with greater accuracy.

### 2.2 Steering the Particle

Most of the current work on OT moves the laser trap position to the desired destination of the particle and lets the particle drift to the laser position. For light particles or strong lasers, there would be little difference between the center of the potential field and the particle. But for heavy particles with low power lasers, there will be noticeable time delay between the two positions. It is much preferable to use lower power lasers for many reasons other than being more economical. For example, strong lasers can damage the exposed particle. It is also true that the attraction force is not maximum at the center point. By putting the particle in the maximum force region, the steering response time can be reduced. When the particle needs to be moved quickly, the particle is put in these regions to reduce the response time.

With the use of the fourth-order Runge-Kutta method, the system of differential equations in Eq. (6) can be converted to difference equations,

$$\begin{array}{l} \Delta x=\frac{1}{6}\Delta t\cdot \dot{x}+\frac{1}{3}\Delta t\left(\dot{x}-\frac{\log(2)\Delta tP_{0}x}{ma^{2}}e^{-\ln(2)\left(\frac{x^{2}}{a^{2}}+\frac{y^{2}}{b^{2}}\right)}\right)+\\ \frac{1}{3}\Delta t\left[\dot{x}-\frac{\log\left(2\right)\Delta tP_{0}\left(x+\frac{1}{2}\Delta t\dot{x}\right)}{ma^{2}}e^{-\ln(2)\left(\frac{{\left(x+\frac{1}{2}\Delta t\dot{x}\right)}^{2}}{a^{2}}+\frac{y^{2}}{b^{2}}\right)}\right]+\\ \frac{1}{6}\Delta t\left(\dot{x}-\frac{2\log\left(2\right)\Delta tP_{0}\left[x+\frac{1}{2}\Delta t\left(\dot{x}-\frac{\log\left(2\right)\Delta tP_{0}x}{ma^{2}}e^{-\ln\left(2\right)\left(\frac{x^{2}}{a^{2}}+\frac{y^{2}}{b^{2}}\right)}\right)\right]}{ma^{2}}e^{-\ln\left(2\right)\left\{\frac{{\left[x+\frac{1}{2}\Delta t\left(\dot{x}-\frac{\log\left(2\right)\Delta tP_{0}x}{ma^{2}}e^{-\ln\left(2\right)\left(\frac{x^{2}}{a^{2}}+\frac{y^{2}}{b^{2}}\right)}\right)\right]}^{2}}{a^{2}}+\frac{y^{2}}{b^{2}}\right\}}\right)\\ \Delta y=\frac{1}{6}\Delta t\cdot \dot{y}+\frac{1}{3}\Delta t\left(\dot{y}-\frac{\log\left(2\right)\Delta tP_{0}y}{mb^{2}}e^{-\ln\left(2\right)\left(\frac{x^{2}}{a^{2}}+\frac{y^{2}}{b^{2}}\right)}\right)+\\ \frac{1}{3}\Delta t\left[\dot{y}-\frac{\log\left(2\right)\Delta tP_{0}\left(y+\frac{1}{2}\Delta t\dot{y}\right)}{mb^{2}}e^{-\ln\left(2\right)\left(\frac{x^{2}}{a^{2}}+\frac{{\left(y+\frac{1}{2}\Delta t\dot{y}\right)}^{2}}{b^{2}}\right)}\right]+\\ \frac{1}{6}\Delta t\left(\dot{y}-\frac{2\log\left(2\right)\Delta tP_{0}\left[y+\frac{1}{2}\Delta t\left(\dot{y}-\frac{\log\left(2\right)\Delta tP_{0}y}{mb^{2}}e^{-\ln\left(2\right)\left(\frac{x^{2}}{a^{2}}+\frac{y^{2}}{b^{2}}\right)}\right)\right]}{mb^{2}}e^{-\ln\left(2\right)\left\{\frac{x^{2}}{a^{2}}+\frac{{\left[y+\frac{1}{2}\Delta t\left(\dot{y}-\frac{\log\left(2\right)\Delta tp_{0}y}{mb^{2}}e^{-\ln\left(2\right)\left(\frac{x^{2}}{a^{2}}+\frac{y^{2}}{b^{2}}\right)}\right)\right]}^{2}}{b^{2}}\right\}}\right) \end{array}$$ (7)

where ∆*x* and ∆*y* are the displacements of the particle in the *x*, *y* directions. Equation (7) computes the displacement of a particle due to the potential field in ∆*t*. Inversely, with known ∆*t* and the velocity of the particle, one can compute the location of the particle *x*, *y* relative to the center of the potential field. In other words, the center of the potential field relative to the particle location that would move the particle in ∆*t* time with the displacement of (∆*x*, ∆*y*) is –*x*, –*y*. To solve for *x*, *y* in Eq. (7) Newton's method [10] is used. The success of Newton's method depends on providing it with a sufficiently good initial guess. The initial guess used is obtained in the parameter domain *x* and *y*. The domain is first divided into square elements. Now for each element, the linear segments of the curve satisfying the first part (∆*x*) of Eq. (7) is computed. The linear segments are determined by the signs of ∆*x* at four corner points. This technique is a specialization of the Marching Cubes algorithm [11] applied to two dimensions. For each element with segments satisfying ∆*x*, the second segment set satisfying ∆*y* is obtained once again. At the end, the possible intersection points between the first and the second sets are computed. These intersection points are refined through Newton's method. If the Newton's method does not converge, the element is subdivided into smaller sub elements, and the procedure is started all over again.

### 2.3 Working Envelope

The working envelope (WE) is defined as the envelope of displacement vectors a particle can be moved by a potential field. The WE is dynamic in that it changes due to the velocity of the particle and the time lapse until the next control cycle. WE limits the motion of the particle.

The WE is numerically computed. By using the coordinate values at equally spaced, regular grid points as the displacement vectors of a particle with certain elapsed time and initial velocity of the particle, solutions to Eq. (7) are sought. The grid points where solutions exist form a lump. By connecting the boundary of this lump with a loop composed of line segments, one gets the WE. Feldman's algorithm [12] is used for computing the loop pruning the spurious branches. Furthermore, the entire interior set of grid points to the loop are checked to determine if they have solutions. Each point with no solution is recalculated with smaller square elements for computing the initial guess point to see if that would lead to solutions. In cases where this recursive refinement does not eventually lead to a solution, an average of neighboring solutions is used as the solution.

### 2.4 User Interface

The operator steers the particle with a cursor, which is a small spherical ball that is position controlled by the stylus of the haptic device. The operator moves the cursor to the particle of interest and presses the button attached to the stylus and then the particle is rigidly glued to the cursor. The system is now in the steering mode. The system then shows the WE of the particle in question. The WE depends on the time lapse between the user control cycles and the velocity of the particle. The haptic stylus shows no resistance when the particle is moved inside the WE yet encounters a virtual haptic wall when the particle is moved outside the WE. A simple linear spring model is used to render the wall.

**Fig. 1:**
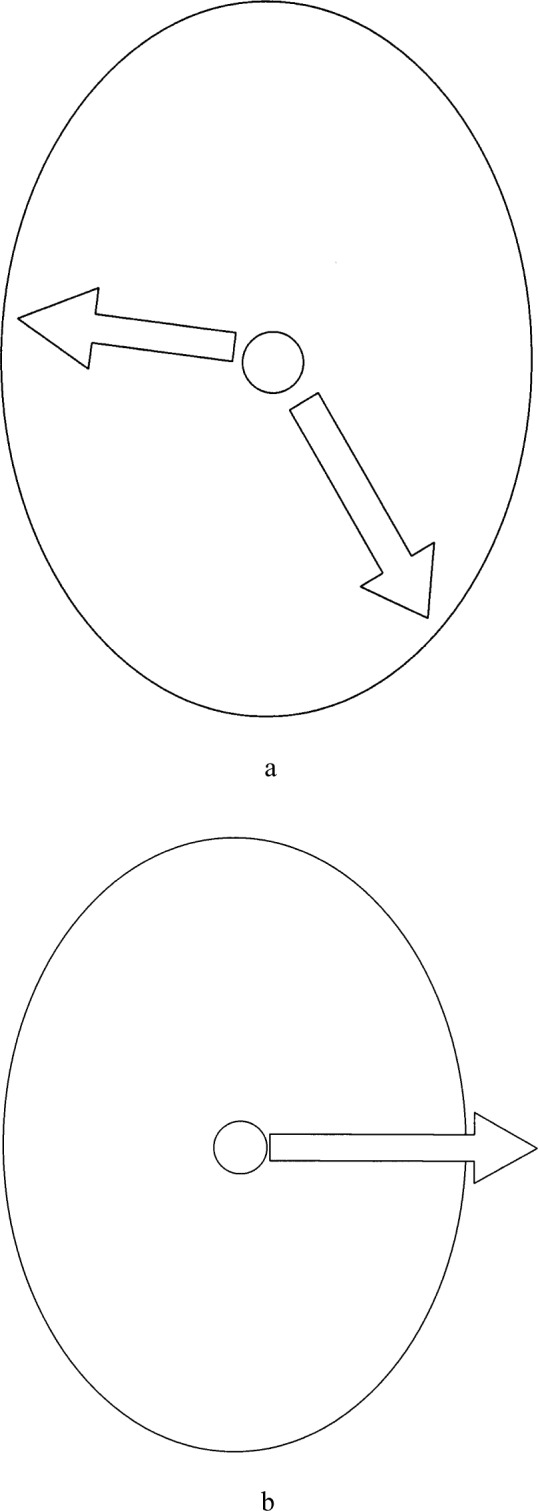
Displacement of the particle (a) within WE (Working Envelope) (b) outside WE (Working Envelope).

The ellipse in Fig. 1 (a) represents the WE of the particle at the center. When the commanded displacement of the particle is within the boundary of the WE, such as the two arrows that are inside the ellipse, the operator experiences no reaction force, and the particle is moved to that position indirectly by the movement of the potential field. In Fig. 1 (b), however, the commanded displacement is outside the boundary of the WE. In this case the displacement intersects with the WE and the particle is moved up only to the intersection point. The excess amount of the displacement command (initial displacement command subtracted by the modified displacement command) is used to determine the relative magnitude of the reaction force. This way the operator would sense a virtual wall at the WE. Notice this way the particle would still move when the issuing command is physically incorrect. This maximizes the capability of the optical tweezers.

## 3. Results

The results are divided into three subsections. Firstly, understanding of the particle trajectory is gained with various experiments. Secondly, the numerical results in computing the laser position and WE is given. Lastly, the virtual environment is presented.

### 3.1 Characteristics of the Particle Movement

**Fig. 2:**
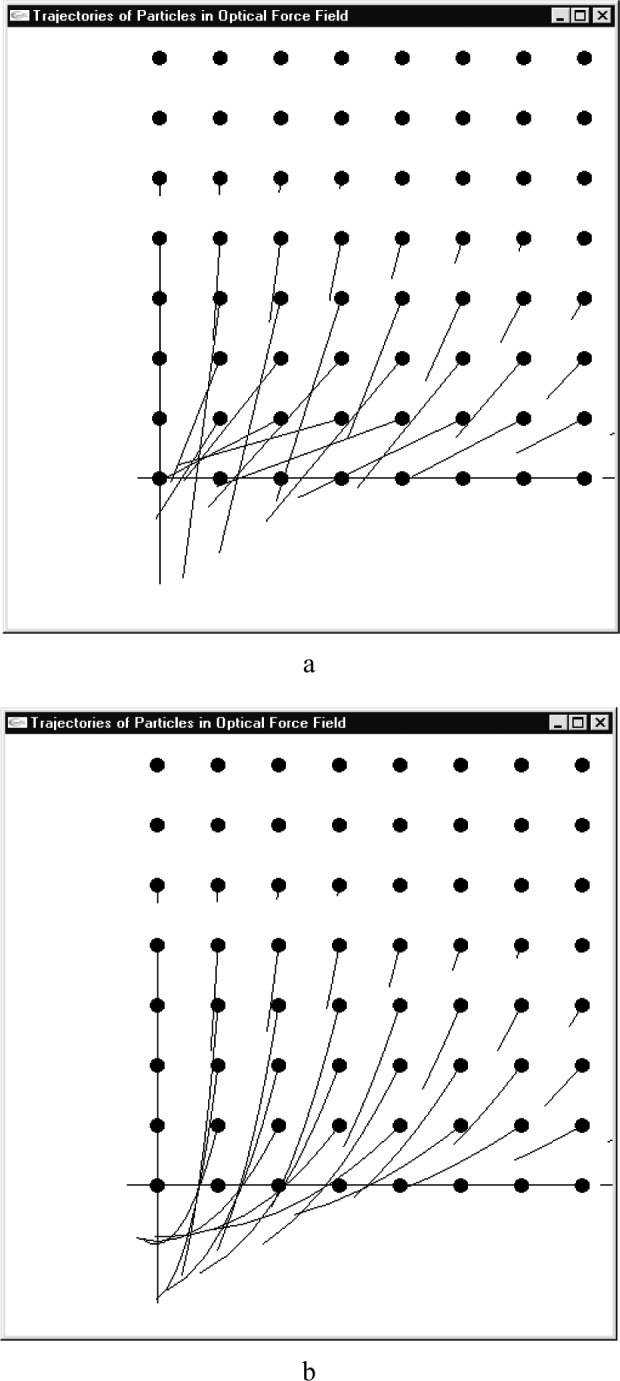
Trajectories of particles in optical potential field (a) one time step of 1 μs (b) ten time steps of 0.1 μs.

The trajectories of particles in the potential field can be computed by Eq. (7). To gain insight into how the particles would move in the potential field, the trajectories of particles placed in grid points starting from rest is plotted. The result is illustrated in Fig. 2 (a). Following constants are used: *a* = 600 nm, *b* = 300 nm, *p*_0_ = 1.0×10^–6^ nJ, *m* = 1.0×10^–12^ g, *ẋ* = 0 nm/μs, *ẏ* = 0 nm/μs and ∆*t* = 1.0 μs. The grid spacing in Fig. 2 is 160 nm. To examine the nonlinear nature of the trajectory, the experiment is tried with multiple time steps. The result of using 10 time steps totaling one μs is given in Fig. 2 (b). It can be seen that a more accurate result can be achieved by using multiple time steps, but it requires more computation time. As mentioned before, fourth-order Runge-Kutta with a fixed time step was used for the numerical integration. The calculated results agreed with the results from the commercial numerical solver (SIMULINK^1^). A varied time step method was also tested in SIMULINK giving agreeing results.

**Fig. 3:**
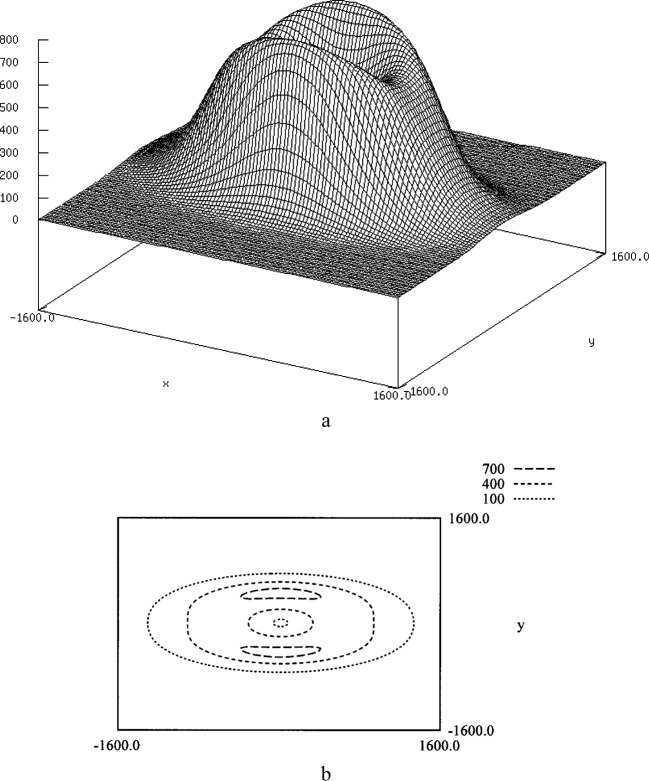
Plot of displacement distances (a) magnitude (b) iso-contours of equal distances.

**Fig. 4:**
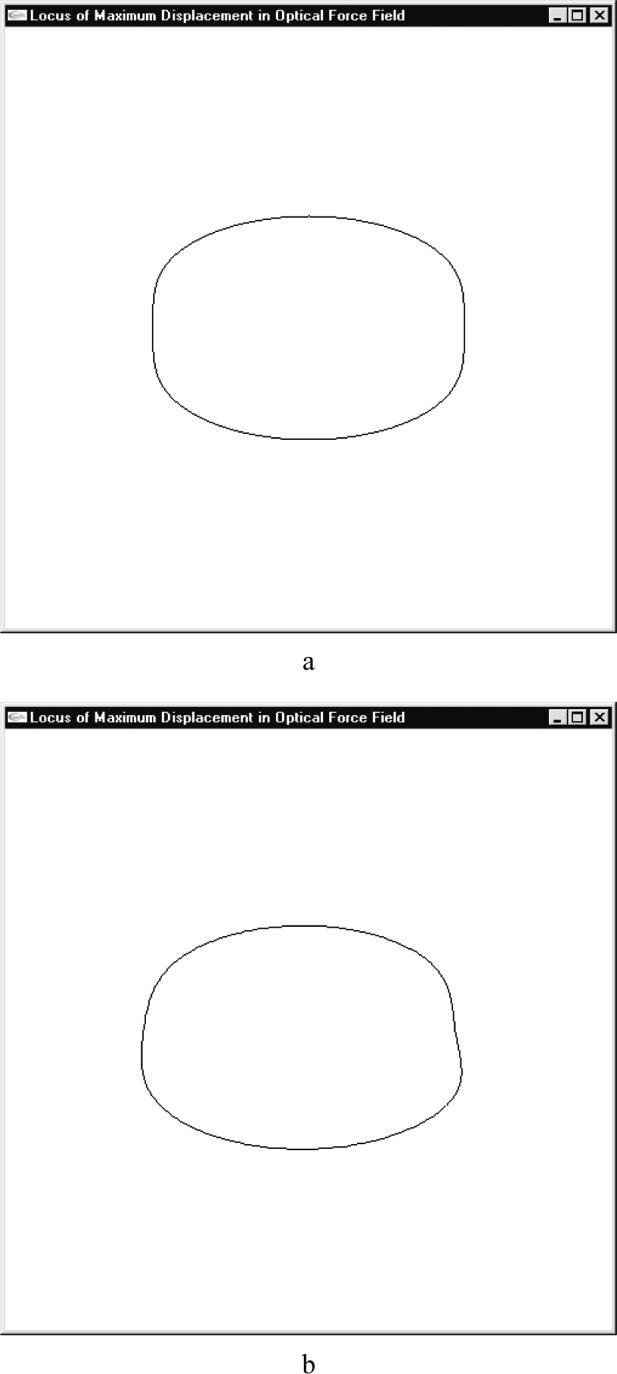
Locus of maximum displacements in radial directions (a) particle initially at rest (b) particle initially moving.

The next question was how far the particles would travel in the given potential field. Figure 3 (a) is a plot of the displacement distance as a function of the particle location from the center of the potential field. Figure 3 (b) is the plot of iso-contours having the same displacement distance. The legend of the distances is shown at the top right corner. Notice that two spots symmetrical to the horizontal axis have maximal displacement. These spots can be important as they dictate the displacement limit of a given laser force field. It is also important to note that the center spot of the laser beam shows low displacement distance. The locus of maximal displacements in radial directions is given in Fig. 4 (a). The directions were sampled uniformly in increments of 3.6°.

In realistic situations the particles would have velocities. The effect of velocities on the particle trajectory is studied. In Fig. 4 (b), the following constants are used: *ẋ* = 100 nm/μs, *ẏ* = 100 nm/μs. Notice the locus is changed due to the initial velocity of the particle.

**Fig. 5:**
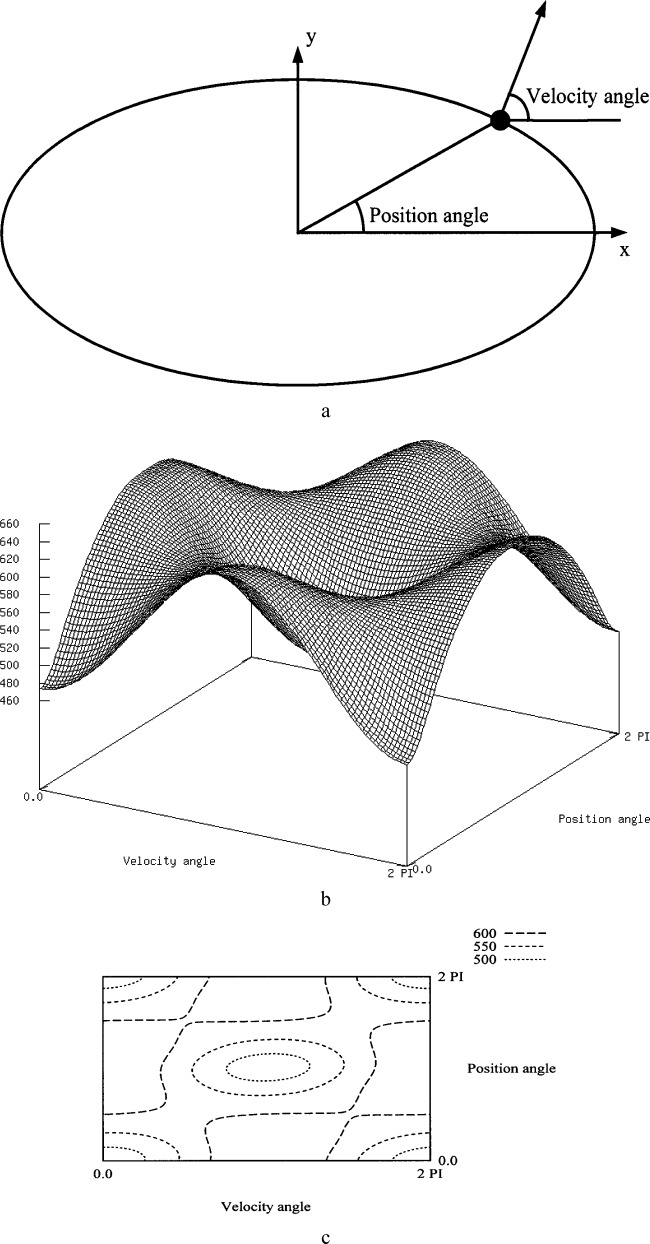
Plot of displacement distances for constant energy (a) coordinate system (b) magnitude (c) iso-contours of equal distances.

In Fig. 3, the particle was initially at rest, the kinetic energy being zero. Since the potential energy is higher on the periphery than at the center, the combined total energy is higher on the periphery. This means that each (*x*, *y*) location in Fig. 3 depicts a particle in a different total energy state. One can obtain a different plot when one enforces constant total energy. Fig. 5 (a) is the coordinate system used for the plot in Fig. 5. The ellipse denotes the constant potential energy curve. The small black circle lying on the ellipse represents a particle with a velocity at a certain angle (“velocity angle”) to the horizontal axis. The position on the curve is determined by the “position angle.” Angles in Fig. 5 (b) and (c) are shown in radians. The particle displacement distance is plotted by varying the particle position and its velocity direction on a constant potential energy (1.0×10^–6^ nJ) curve with constant kinetic energy (5.0×10^–9^ nJ). The result is shown in Fig. 5 (b). Figure. 5 (c) is the plot of iso-contours having the same displacement distance.

### 3.2 Computing the Laser Position

**Fig. 6:**
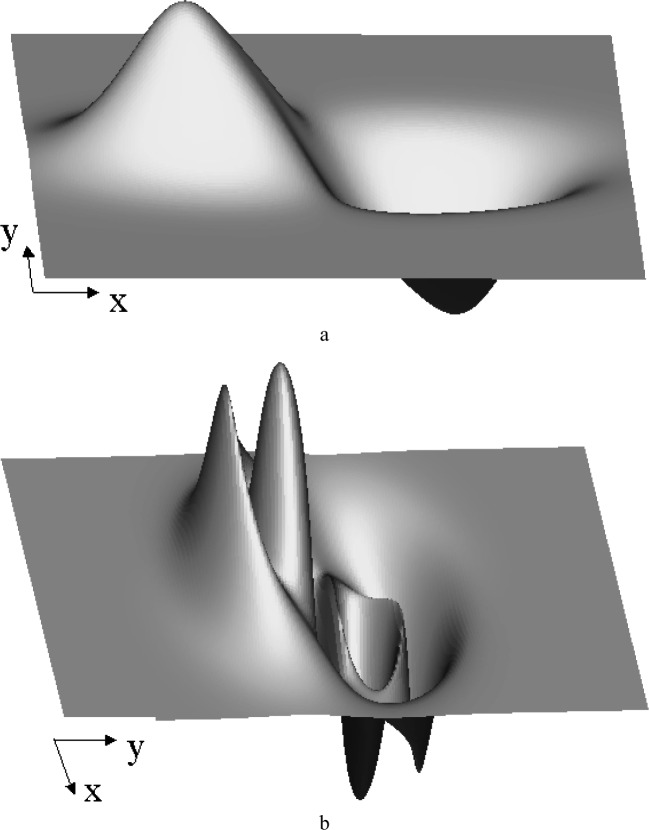
Solution surface (a) plot of ∆*x* (b) plot of ∆*y*.

**Fig. 7:**
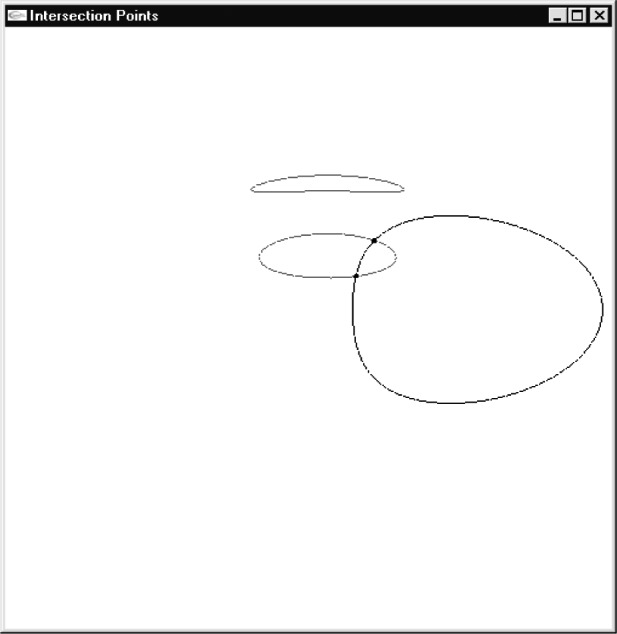
Solution points.

**Fig. 8:**
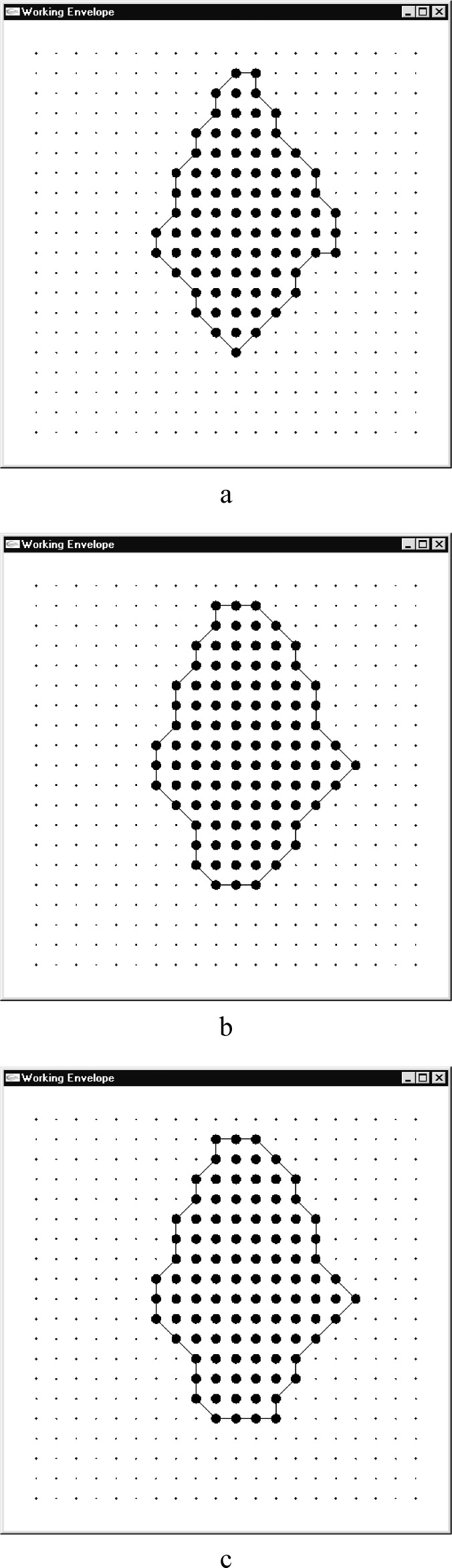
Working envelope (a) 20×20 grid points used for computing the initial guess point (b) 80×80 grid points used for computing the initial guess point (c) 320×320 grid points used for computing the initial guess point.

Now comes the most important part, computing the center of the potential field such that a particle at a certain initial velocity would displace to a required position in a given amount of time. The solution of Eq. (7) can be very involved due to the highly nonlinear nature of ∆*x* and ∆*y*, especially when ∆*t* is large. The plots of ∆*x* and ∆*y* are given in Fig. 6 (a) and (b), respectively. Following parameters are used: ∆*t* = 1.3 s, *ẋ* = 100 nm/μs, *ẏ* = 100 nm/μs. For visual clarity the axes are moved to the bottom left. The origin is at the center. In Fig. 7, the two small loops aligned vertically at the center are the locus of points satisfying ∆*y* = –800 nm. Similarly, the biggest loop is the locus of points satisfying ∆*x* = –160 nm. The two intersection points shown in Fig. 7 are the two solution points satisfying both conditions. If this is extended spatially, and the solutions are sought, one can compute discrete points where the solutions exist or do not exist. For example, when one uses the coordinates of regular grid points as ∆*x*, ∆*y* and use small dots to represent points where there are no solutions and larger dots for points where there are, one can get Fig. 8 (a). The initial guess points were obtained with the grid distance equally spaced as shown in Fig. 8 (a). The computation took 17 s on a Pentium PC. Notice that the envelope of solvable grid points is connected with line segments. The connected segments form the WE. If desired, one can get more accurate results by using a finer grid distance for the initial guess points. Figure 8 (b) was obtained with four times finer resolution and took 288 s. Furthermore, Fig. 8 (c) was obtained with sixteen times finer resolution and took 4591 s. Notice there is very little difference between Fig. 8 (b) and Fig. 8 (c). Formal formulation for optimal grid spacing for the initial guess point is not easy to do. Therefore the results as a function of different resolutions are examined and the one where the increase in the resolution did not significantly improve the solution was chosen.

### 3.3 The Virtual Environment

**Fig. 9:**
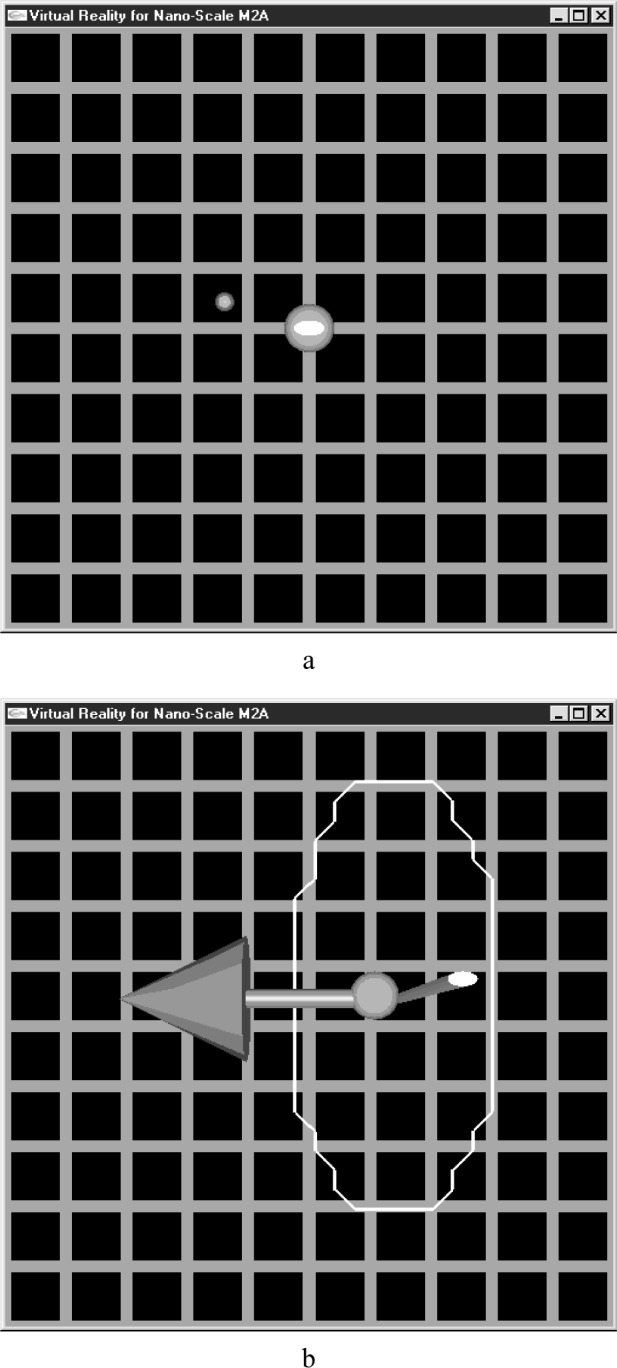
(a) Initial screen of the virtual environment (b) the reaction force experienced by over running the particle.

Figure 9 (a) illustrates the initial screen of the virtual environment (VE). The descriptions of the graphical objects are as follows. The solid sphere at the center is the particle. It is being illuminated by a dithered laser beam shooting in the direction of the plane. This is shown as the ellipse in Fig. 9 (a). The small sphere to the left of the particle is the cursor for the operator. When the haptic stylus is moved, the cursor follows accordingly. The particle can be moved only after it has been grabbed. To grab the particle, the operator moves the cursor to either touch or be inside the particle and presses the button attached to the stylus. To release the particle the operator simply releases the button. The diameter of the particle is one μm and the grid spacing distance is 2.9 μm. The physical parameters used for the potential field are *a* = 600 nm, *b* = 300 nm, *p*_0_ = 1.0×10^–6^ nJ, *m* = 1.0×10^–12^ g.

Figure 9 (b) illustrates the instant snap shot when the particle is steered to the right and the displacement amount exceeds the physical capability of the potential field. The virtual environment reacts by reacting with a force that is in the direction counter to the steering motion. The amount of force is proportional to the exceeding distance from the WE. The PHANTOM haptic device was used for the haptic output and steering. This was attached to a Windows NT with a Pentium processor and 256 MB of RAM. The descriptions of the graphical objects are as follows. The arrow direction and length shows the direction and magnitude of the reaction force applied to the haptic stylus. The slender rod with elliptical cross section denotes the dithered laser beam. Finally the closed loop shows the WE. Because the WE is too small, it was drawn with a scale factor of 100. The WE and its interior solution points are pre-computed before the execution. The bounding box region with range of –100 nm to 100 nm on each axis was used to compute the WE. The cached data depends upon the elapsed time and the initial velocity of the particle. For simplicity and the limitation of the computer memory, a constant cycle time of 0.2 μs was used. The initial velocity of the particle at each axis was varied from –80 nm/μs to 80 nm/μs in increments of 5.33 nm/μs. To introduce a viscous effect and also to narrow down the range of velocity of the particle to be considered, the velocity was reduced to one tenth of the preceding cycle at each cycle. Note that a more formal way to have done this would be to have modeled the viscous force as a function of a velocity in Eq. (6). This would have changed the dynamics described in Eq. (6) and Eq. (7). It was not done in this way for simplicity. The total processor time to compute this in a batch process using an SGI Onyx2 with four processors and 1 GB of main memory took 71 487.99 s (about 20 h).

## 4. Discussion

The discreteness of the WE introduced some jaggedness to the haptic sensation. Recall that the method of computing the WE used grid points and the smoothness of the WE is directly proportional to the grid distance. The smaller the grid distance, the smoother is the WE. However, the use of force feedback for constraining the steering motion of the operator that exceeded the laser capability did prove to be a useful method. The reaction force from the haptic device made it natural for the operator to slow down. In one experiment with the haptic feedback off, the distance between the operator cursor and the particle reached up to 3 μm. In another experiment with the haptic feedback on, the distance between the operator cursor and the particle was kept under 0.3 μm. In order to use this VE in tele-operation, much research needs to be done to understand the interaction between the laser beam and the particle and also between the particle and the surrounding fluid. Validating the model requires deriving a more detailed mathematical model for the potential along with extensive experiments. Also the viscous effect of the surrounding fluid needs to be taken care of. When the particle is not a simple sphere, as used in this article, the orientation of the particle needs to be considered when computing the WE. This means two more variables to represent the orientation need to be added to the initial three variables (elapsed time, initial velocity in the *x* and *y* directions).

When the problem domain is expanded to three dimensions, it is presumed that most approaches used in two dimensions could be used without major changes, with several exceptions. The method for computing the initial guess point for Eq. (7) cannot be extended to three dimensions and needs a new numerical method. Also the memory requirement can be substantial in three dimensions. The cached data for the WE uses 28 MB. An Added dimension (*z* axis) would easily use 1 GB. For a nonspherical geometry one would also need to add two more dimensions for the orientation. Obviously, a more memory efficient representation of the WE needs to be studied.

## 5. Conclusion

Optical tweezers are a new device that has great potential for manipulating nanoscale objects. The non-intrusive nature of the device enables nondestructive manipulation. To better understand the underlying physical nature of the device, a virtual environment that can simulate the physics of the laser beam and particle interactions has been designed and implemented. The conversion of the system of differential equations to a system of difference equations enabled the precise control of the transient position of the particle. By putting the particle in the maximum force region, the maximum movement with the given laser condition was achieved. A new concept called WE (Working Envelope) and a complex numerical technique to compute it was also presented. It was shown that WE can prevent the user from attempting physically impossible movement of the particle.

The simulation environment could have more value when it is used in a tele-operation environment. One would have finer position control without ever losing the grip. This requires a more sophisticated potential model and experiments.

## Footnotes

1 Certain commercial equipment, instruments, or materials are identified in this paper to foster understanding. Such identification does not imply recommendation or endorsement by the National Institute of Standards and Technology, nor does it imply that the materials or equipment identified are necessarily the best available for the purpose.
